# EARLY LIFE INFLUENCES ON LATER LIFE ADRD RISK: THE ROLES OF ADOLESCENT COGNITIVE FUNCTION AND EDUCATION

**DOI:** 10.1093/geroni/igae098.1355

**Published:** 2024-12-31

**Authors:** Pamela Herd, Sanjay Asthana, Michal Engelman, Kamil Sicinski, Ralph Trane, Victoria Williams

**Affiliations:** Georgetown University, Washington, District of Columbia, United States; University of Wisconsin Madison, Madison, Wisconsin, United States; University of Wisconsin Madison, Madison, Wisconsin, United States; University of Wisconsin Madison, Madison, Wisconsin, United States; University of Wisconsin Madison, Madison, Wisconsin, United States; University of Wisconsin Madison School of Medicine and Public Health, Madison, Wisconsin, United States

## Abstract

By some estimates, each additional year of education reduces dementia risk by 7 percent. What remains unclear, however, is how early life cognitive function, which influences educational attainment, and is hypothesized to influence later life dementia risk, factors into the relationship between education and dementia risk. Existing research neither attends to the role that early life cognition may play in these relationships, nor does it separate out how these relationships might vary with AD versus non-AD dementias. We employ a unique dataset, the Wisconsin Longitudinal Study, which has been tracking participants over their full life course and is now assessing dementia in the population as they reach their mid 80s. The data include prospectives measures of early life cognition, other early life conditions, as well as detailed educational histories, in addition to dementia diagnoses that differentiate AD and non-AD types. We find that lower early life cognitive function increases the risk for AD dementia, and education does not mediate this relationship. Early life cognition, however, has no relationship with non-AD dementia, that is dementia caused by chronic disease, where instead education has a stronger relationship. We hypothesize that these differential relationships reflect variance in how early life biological and environmental factors affect risk and resilience for later life cognitive dysfunction.

